# SCUD: *Saccharomyces Cerevisiae *Ubiquitination Database

**DOI:** 10.1186/1471-2164-9-440

**Published:** 2008-09-24

**Authors:** Won-Chul Lee, Minho Lee, Jin Woo Jung, Kwang Pyo Kim, Dongsup Kim

**Affiliations:** 1 Department of Bio and Brain Engineering, Korea Advanced Institute of Science and Technology, Daejeon 305-701, Korea; 2 Department of Molecular Biotechnology, Konkuk University, Seoul 143-701, Korea; 3 Institute of Biomedical Science and Technology, Konkuk University, Seoul 143-701, Korea

## Abstract

**Background:**

Ubiquitination is an important post-translational modification involved in diverse biological processes. Therefore, genomewide representation of the ubiquitination system for a species is important.

**Description:**

SCUD is a web-based database for the ubiquitination system in *Saccharomyces cerevisiae *(Baker's yeast). We first searched for all the known enzymes involved in the ubiquitination process in yeast, including E1, E2, E3, and deubiquitination enzymes. Then, ubiquitinated substrates were collected by literature search. Especially, E3 and deubiquitination enzymes are classified into classes and subclasses by their shared domains and unique functions. As a result, 42 different E3 enzymes were grouped into corresponding classes and subclasses, and 940 ubiquitinated substrates including mutant substrates were identified. All the enzyme and substrate information are interconnected by hyperlinks, which makes it easy to view the enzyme-specific ubiquitination information.

**Conclusion:**

This database aims to represent a comprehensive yeast ubiquitination system, and is easily expandable with the further experimental data. We expect that this database will be useful for the research on the ubiquitination systems of other higher organisms.

SCUD is accessible at

## Background

Ubiquitination is involved in many biological processes including cell signaling pathway and protein quality checkpoint system, by which ubiquitinated proteins are delivered to the 26S proteasome and degraded. Many recent studies have demonstrated that protein ubiquitination not only guarantees its degradation but also results in other consequences such as receptor endocytosis and protein transport, indicating that ubiquitination is an important process with highly complicated biological behaviors.

Experimental results about ubiquitination of numerous substrates have been accumulated, but there is no database which can easily facilitate further experiments or computational analysis. Recently, a database, UbiProt, which contains the information about ubiquitinated substrates from diverse organisms, has been appeared [1]. However, UbiProt is highly dependent on a small number of high-throughput proteomics studies. Moreover, it fails to include many previously identified substrates. Importantly, UbiProt has little information about enzymes which are involved in the ubiquitination process.

Ubiquitination of a substrate is carried out by sequential reactions of enzymes. First, E1 ubiquitin-activating enzyme modifies a ubiquitin so that it is activated to a reactive state (making it likely that C-terminal glycine on the ubiquitin will react with the lysine side-chains on the substrate protein). Then, E2 ubiquitin-conjugating enzyme receives an activated ubiquitin, and catalyzes the attachment of the ubiquitin to the substrate protein in concert with E3 ubiquitin-protein ligase. Here, E3 enzymes are important as they determine the specificity to their substrates. Either a protein complex or a unit protein plays a role as E3 ligase. In case of E3 ligase complex, a specialized subunit protein called substrate receptor performs docking with the substrates, while the unit E3 ligase has a special domain with which the substrates interact. Therefore, it is necessary to identify all the known E3 enzymes, followed by classifying them according to their unique domain or complex type, and finally discovering E3-specific ubiquitinated substrates. If we are able to distinguish the substrates by E3 enzymes, it is possible to find sequence motifs or structural characteristics that contribute to the E3 enzyme-substrate specific interaction. Moreover, ubiquitinated site information would help identify the conditions on which specific lysines are selected and ubiquitinated. For these purposes, we searched for all the known enzymes involved in the ubiquitination process in budding yeast, *Saccharomyces cerevisiae*, and classified E3 enzymes into E3 classes and subclasses. Then, we collected ubiquitinated substrates either by E3-based literature search or by reference to high-throughput proteomics studies. This database aims to represent a comprehensive ubiquitination system in yeast.

## Construction and content

We chose yeast as a model organism because a majority of known ubiquitination substrates were obtained from yeast. Moreover, the ubiquitination enzyme system is highly sophisticated and has many unrevealed aspects in other higher organisms, especially in human, making it difficult to systematically represent the enzymes involved in the ubiquitination process. Therefore, the yeast ubiquitination system appears a good platform from which the ubiquitination research can be extended to other organisms.

### Identification of Ubiquitination Enzymes and E3 enzyme Classification

We searched for all known ubiquitination enzymes including E1, E2, and deubiquitination enzymes through Saccharomyces Genome Database (SGD, ) and Swiss-Prot . For E3 enzymes, we looked for all the proteins showing ubiquitin-protein ligase activity and extracted the proteins involved in substrate recognition independently or dependently on other proteins (in case of complexes). We called these proteins 'substrate receptors' that can act as a substrate-recognition subunit of E3 ligase complex or as E3 ligase alone. It has been known that E3 enzymes can be divided into different groups. We classified the substrate receptors according to the following E3 enzyme classes and subclasses.

● HECT (Homologous to E6-AP C terminus) class [2]

● RING (Really Interesting New Gene) class [3,4]

■ SPRF (Single Protein RING Finger) subclass: Single protein ligases containing RING domain. They possess both the substrate recognition part and the E2 interaction part on them.

■ SCF (SKP1, CDC53, and F-box) subclass: Cullin (CDC53)-dependent multi-subunit E3 enzyme complex. F-box proteins are in charge of substrate recognition.

■ ECS (ELC1, CUL3, and SOCS/BC-box) subclass: Cullin (CUL3)-dependent multi-subunit E3 enzyme complex. SOCS/BC-box proteins are involved in substrate recognition.

■ U-BOX subclass: U-BOX is similar to RING domain.

■ APC (Anaphase Promoting Complex) subclass: Multi-subunit E3 enzyme complex. Its subunit "APC" is similar to Cullins in SCF and ECS complex.

**Figure 1:**
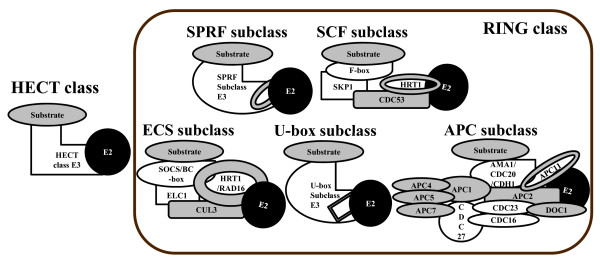
Diagrams of E3 enzyme classes and subclasses.

Here, U-box containing proteins were previously known as E4 enzyme which promotes the polyubiquitination [5]. However, it has been also verified that U-box proteins can intermediate the substrate ubiquitination with E1 and E2, regardless of E3 involvement. This results in defining the U-box proteins as a new family of ubiquitin ligases [4]. Diagrams of HECT class and 5 RING subclasses are described in Figure 1.

### Identification of Ubiquitinated Substrates

Using each substrate receptor in each E3 enzyme class or subclass as a query, we collected by literature search all substrate information as well as E2 and deubiquitination enzymes which participate in the substrate ubiquitination. The substrate information includes the ubiquitination site, type, enzymes, and ubiquitination effect as well as related references. In addition, we included the ubiquitinated substrates identified by several proteomics approaches [6-9], although most of them do not have the specific information on ubiquitination enzymes.

On the other hand, we also found the mutant and synthetic substrates which are ubiquitinated by already known E3 enzymes. In the endoplasmic reticulum (ER), two representative E3 enzymes (HRD1 and SSM4) are responsible for the ubiquitination of most ER substrates [10]. HRD1 and SSM4 target the misfolded mutant ER proteins as well as wild-type proteins. Moreover, UFD (Ubiquitin Fusion Degradation) pathway protein UFD2 and UFD4 are involved in the ubiquitination of synthetic ubiquitin fusion proteins such as Ub^V76^-V-βgal, while their physiological substrates are mostly unknown [11].

### Database Content

**Figure 2:**
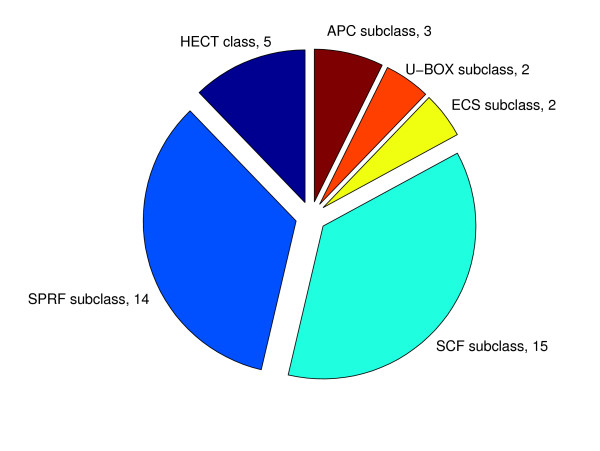
**The numbers of E3 enzymes for each E3 class and subclass**. Total 42 E3 enzymes are sorted into different E3 classes and subclasses.

We found total 1 E1, 11 E2, 42 E3, 20 deubiquitination enzymes, and 940 ubiquitinated substrates including mutant and synthetic substrates (see Additional file 1). The E3 enzymes are further grouped into different E3 classes and subclasses (Figure 2). Most E3 enzymes possess their own substrates, while the substrates of some E3 enzymes are not known yet.

## Utility and discussion

**Figure 3:**
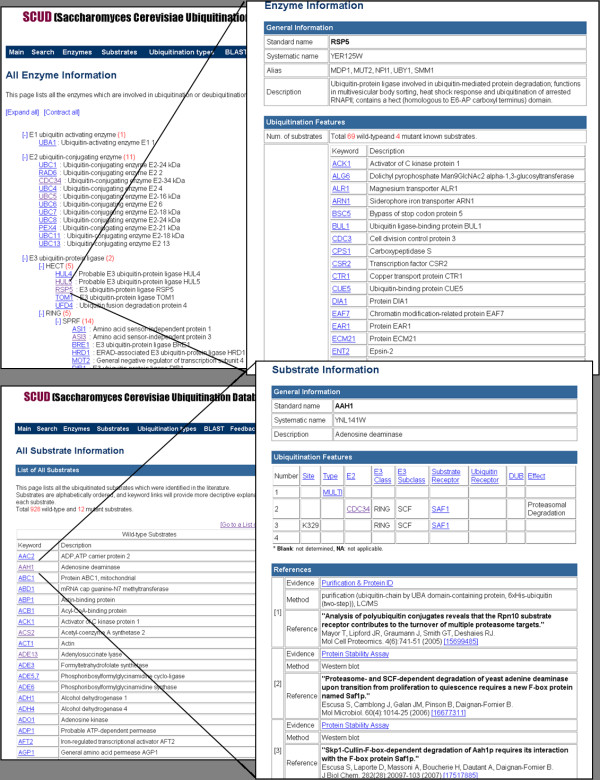
**Enzyme and substrate information**. Lists of all known enzymes and substrates. Hyperlinks provide detailed information on individual enzymes and substrates.

The interfaces of our database were implemented using MySQL, PHP, AJAX, and Python. The database displays a list of all known E1, E2, E3, and deubiquitination enzymes found in the yeast ubiquitination system. Particularly, E3 and deubiquitination enzymes are grouped based on their shared domains or distinct functions. The detailed information on an individual enzyme is provided by a hypertext link. It includes general protein information as well as a list of substrates ubiquitinated by the enzyme (Figure 3). Most E3 enzymes possess their own substrates, while the substrates of some E3 enzymes are not known yet. Similarly, a list of all known substrates and individual substrate information are also available. The individual substrate information involves ubiquitination site, type, enzymes, receptors, effect, and general protein information (Figure 3). All the enzymes and substrate information are associated by hyperlinks.

On the other hand, it is necessary to provide the experimental evidences by which proteins are regarded as ubiquitin-conjugates to ensure the quality of identified substrates. For example, it is well known that ubiquitin-modified proteins are likely to be degraded by proteasome or vacuole. Therefore, sometimes proteins showing stability change are assumed to be ubiquitinated. However, the stability change can be explained by other reasons except for the ubiquitination. To offer the evidences of ubiquitination, we collected all the experimental evidences of ubiquitinated proteins in the references, categorized them, and included in the database along with detailed methods and related reference information (Figure 3). It will help users to examine the ubiquitinated proteins with their own filtering criterions.

**Figure 4:**
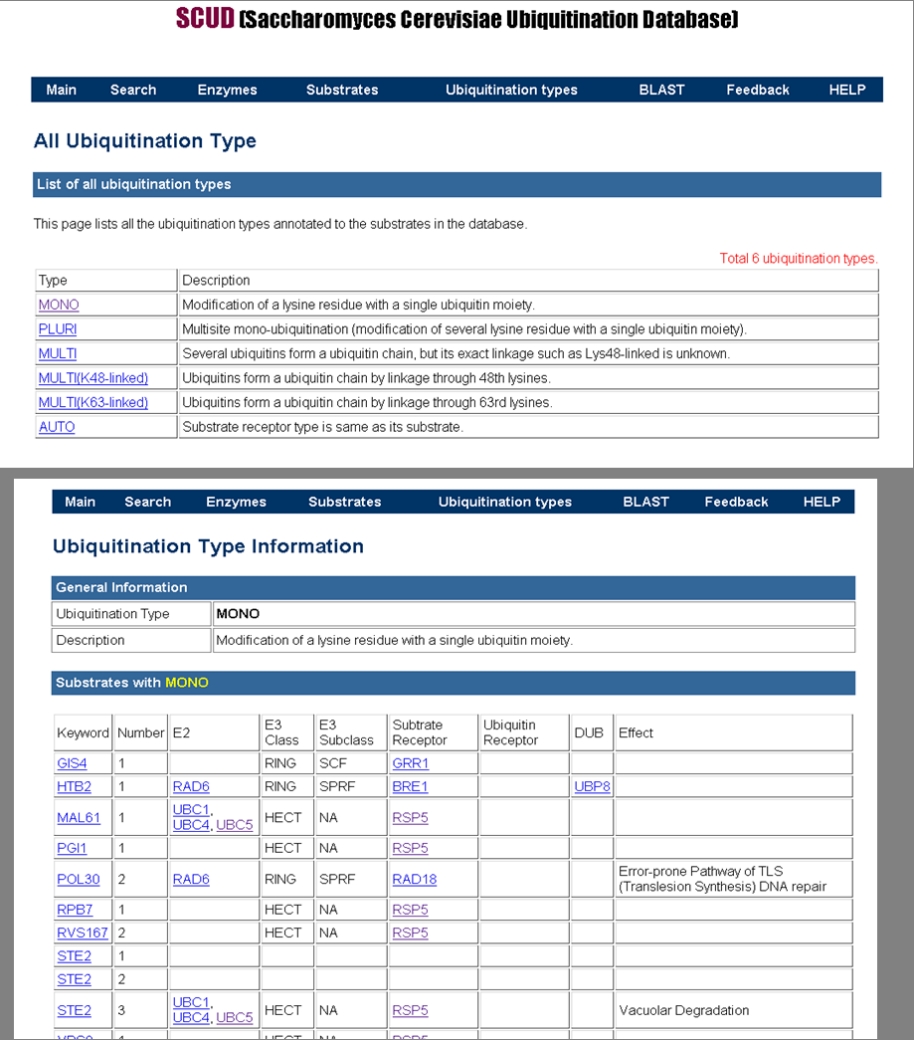
**Ubiquitination types**. A list of all identified ubiquitination types and ubiquitination type-specific information.

Finally, the database enables users to discriminate the substrates by their type of ubiquitination such as mono- and Lys48-linked ubiquitination (Figure 4). We also provide BLAST search service which runs on all the protein sequences including enzymes and substrates in our database. Moreover, researchers in ubiquitination field can contribute to the database by submitting new proteins including enzymes and substrates, by finding errors to be corrected, and by giving general opinions and suggestions about the database itself (feedback session). The database will be regularly updated with additional data and corrections.

## Conclusion

SCUD is a web-based database aiming to represent a comprehensive ubiquitination system for yeast. We identified all known ubiquitination enzymes including E1, E2, E3, and deubiquitination enzymes as well as substrates by literature search. Users can examine various ubiquitination features such as ubiquitination site, type, and effect, and obtain enzyme-specific or ubiquitination type-specific information by hyperlinks. This database is easily expandable with further experimental data. Built-in BLAST search is useful when users want to test putative protein sequences that may belong to the ubiquitination system of yeast or other species. The feedback interface will gather a variety of ubiquitination information as well as opinions and suggestions about the database itself. We expect that it will be useful for the researches on other higher organisms.

## Availability and requirements

SCUD is available at 

License: The database is freely available.

## Authors' contributions

WCL carried out a literature search, participated in database construction, and drafted the manuscript. ML participated in programming for database construction and preparation of the manuscript. JWJ designed an overall database scheme. KPK and DK supervised the work and collaborated in writing the manuscript. All authors have read and approved the manuscript.

## Supplementary Material

Additional file 1 **Ubiquitinated wild-type proteins in SCUD**. Total 928 wild-type substrates with various ubiquitination features such as ubiquitination site, type, enzymes, receptors, and effect. Experimental evidences and detailed methods are also included. Click here for file
